# Quackery and Its Prevention

**Published:** 1908-01-04

**Authors:** 


### Quackery and its Prevention.

The old adage that the people like to be deceived receives no better illustration than can be given by the returns, made at various times by many different investigators, regarding the composition and efficacy of secret and proprietary medicines. So long as human nature remains what it is, so long will the quack flourish and reap rich gain from the credulity of an imaginative public. In the abstract there is no more interesting study for a medical man than the history of quacks and quackery. Confronted with the reality, it is a task beyond the charity of most of us, who are practical medical men before we are idealists and abstract speculative philosophers, to contemplate with equanimity the rise and progress of a sect which numbers Paracelsus and St. John Long among its aristocracy. And in contemplating the reality otherwise than with mild toleration for the deceived, and well weighed justice for the deceivers, the profession is apt to indulge in speculations and anticipations that cannot bear the brunt of open and dispassionate criticism. The wild schemes for the prevention of quackery, and the no less fantastic propositions for measures to ensure the protection of the legitimate practitioner, scarcely need comment here, for most of them have been forgotten by their originators and disowned by their sponsors. The vital question, however, remains: How is it possible to deal with the problem of quackery in a reasonable and equitable manner? And it is a question which earnestly demands consideration by the legislature as well as by the profession.

No country in Europe, it is safe to assert, possesses so large a number of proprietary medicines as England, and in no other country is the traffic in secret remedies so unrestricted. Here, by a curious anomaly of the law, the sale of poisons has been regulated in a praiseworthy degree, while patent remedies have been specifically excluded from the provisions of the Pharmacy Act. Other anomalies and discrepancies the curious may find for themselves in Mr. Cofman's interesting pamphlet, "Secret Remedies and Proprietary Medicines in England and Abroad"—a paper which shows very forcibly the urgent necessity that exists for limiting, by Statute or otherwise, the sale of quack remedies to a trusting and gullible public. No one acquainted with the extent of the trade and the effects of the present indiscriminate sale of such compounds can fail to appreciate that urgent necessity, and take an interest accordingly, in the tentative suggestions that have been made to meet it.

The first of these suggestions is the appointment of a Royal Commission to inquire into and report upon the whole question, and it would appear that the appointment of such a commission is an indispensable preliminary to any action by the legislature. Already in other countries Parliamentary commissions have considered the whole subject and, by pointing out existing anomalies in the Pharmacy Acts and by drawing attention to abuses, have initiated wholesome reforms and effective measures for restricting and regulating the traffic in secret remedies. Royal Commissions do not always fulfil the high expectations of their supporters, but they do, at least, draw the attention of the public to outstanding facts of importance, and their reports furnish at the same time an authoritative basis for future speculation and suggestion. We do not doubt the educative value, so far as the public is concerned, of such reports, and for that reason, recognising as we do that efficient and useful legislation can only be attained when the public as a whole realises as widely and as whole-heartedly as the profession already does, the need for restriction and regulation, we cordially welcome the suggestion. While it is receiving the consideration of the proper authorities, we would draw attention to another suggestion that is worth cordial support. This is that an Anti-Quackery Society, on similar lines to the authoritative body already in existence in Germany, be established. Such a society, by educating the public to recognise the need for reform and amendment, can do much to prepare the way for legislative measures in the future, and if it can capture the lay press to aid it in combating the plague of proprietary remedies its means of usefulness will be hugely increased.

